# Prevalence and Factors Associated With Burnout of Frontline Healthcare Workers in Fighting Against the COVID-19 Pandemic: Evidence From China

**DOI:** 10.3389/fpsyg.2021.680614

**Published:** 2021-08-16

**Authors:** Xin Zhang, Jiahui Wang, Yanhua Hao, Ke Wu, Mingli Jiao, Libo Liang, Lijun Gao, Ning Ning, Zheng Kang, Linghan Shan, Wenfeng He, Yongchen Wang, Qunhong Wu, Wenqiang Yin

**Affiliations:** ^1^Centre of Health Policy & Management, Health Management College, Harbin Medical University, Harbin, China; ^2^Department of Social Medicine, School of Public Health, Health Management College, Harbin Medical University, Harbin, China; ^3^Tong Zhou District’s Volunteer Services Guidance Center of Beijing Municipality, Beijing, China; ^4^Jinyintan Hospital, Wuhan, China; ^5^The Second Hospital Affiliated of Harbin Medical University, Harbin, China; ^6^School of Public Health and Management, Weifang Medical University, Weifang, China

**Keywords:** burnout, COVID-19, healthcare workers, MBI scale, frontline healthcare workers

## Abstract

**Objectives:**

The sudden outbreak of the novel coronavirus disease (COVID-19) plunged healthcare workers (HCWs) into warfare. This study aimed to determine the prevalence of burnout and the factors associated with it among frontline HCWs fighting COVID-19.

**Methods:**

A cross-sectional survey was conducted among frontline HCWs fighting against the COVID-19 in Wuhan, Harbin, and Shenzhen during the period from February 18 to March 4. Finally, HCWs were recruited using cluster sampling, 1,163 HCWs were included in the final analysis. Burnout was measured using a 22-item Maslach Burnout Inventory scale (MBI scale).

**Results:**

Of the participants, 48.6% suffered from burnout, and 21.8% showed a high degree of burnout. Doctors (*b* = 3.954, *P* = 0.011) and nurses (*b* = 3.067, *P* = 0.042) showed higher emotional exhaustion (EE) than administrators. Participants who worked continuously for more than 8 h a day (*b* = 3.392, *P* = 0.000), those who were unable to eat three regular daily meals (*b* = 2.225, *P* = 0.008), whose daily water intake was no more than 800 ml (*b* = 3.007, *P* = 0.000), who slept for no more than 6 h (*b* = 1.609, *P* = 0.036), and who were infected or had colleagues who were infected with COVID-19 (*b* = 4.182, *P* = 0.000) experienced much higher levels of EE, while those who could adhere to infection control procedures (*b* = −5.992, *P* = 0.000), who were satisfied with their hospital’s infection control measures(*b* = −3.709, *P* = 0.001), and who could receive sufficient psychological crisis intervention (*b* = −1.588, *P* = 0.039) reported lower levels of EE.

**Conclusion:**

The study reveals that burnout is prevalent among frontline HCWs and that the known factors associated with burnout, such as workload, and the factors directly associated with COVID-19, such as having insufficient protection, can affect burnout symptoms in frontline HCWs. Synergized and comprehensive interventions should be targeted at reducing its occurrence among frontline HCWs fighting COVID-19.

## Introduction

The novel coronavirus disease (COVID-19) pandemic is a global public health emergency which has greatly impacted health systems and people’s lives worldwide. The COVID-19 pandemic has posed unprecedented challenges to the global health system (Lai et al., 2020), public health laboratories (Corman et al., 2020), hospitals, and critical care departments (Arabi et al., 2020). Healthcare systems and personnel were, for a time, overstretched and overwhelmed. Many frontline healthcare workers (HCWs) have experienced the darkest period of their professional lives. To fulfill their commitment to the responsibilities and obligations of the medical profession, frontline HCWs have done their utmost to rescue the dying and heal the wounded, diagnosing, treating, and nursing COVID-19 patients around the clock. Thus, frontline HCWs fighting against the COVID-19 pandemic have faced severe challenges and have experienced more health problems than non-frontline HCWs, including burnout symptoms, depressive symptoms, and insomnia (Şahin et al., 2020; Serrão et al., 2021), mental health of frontline HCWs fighting against the COVID-19 pandemic is a problem which require attention.

Burnout has been a major concern in the fields of both occupational health and mental health (Wu et al., 2013; Arabi et al., 2020). The outbreak of COVID-19 triggered widespread alarm among HCWs about the potential for burnout. In Asia, according to Li D et al., 34.2% of HCWs from Wuhan Jinyintan Hospital experienced COVID-19 related burnout (Li et al., 2021). Another study developed in China showed that during the COVID-19 pandemic 36.5% of Chinese HCWs experienced burnout (Huo et al., 2021). Matsuo et al. found that the prevalence of burnout among HCWs in Japan during the COVID-19 outbreak was 31.4% (Matsuo et al., 2020). According to Khasne RW et al., more than half (52.8%) of HCWs in India experienced COVID-19-related burnout (Khasne et al., 2020). According to Alsulimani LK et al., the prevalence of burnout among HCWs in Saudi Arabia, which has one of the best healthcare systems in the Middle East (AlHumaid et al., 2020),was 75% during the COVID-19 pandemic (Alsulimani et al., 2021). In Europe, Italy was severely impacted by COVID-19, and Italian HCWs reported relevant work-related burnout symptoms (Barello et al., 2020; Lasalvia et al., 2021). According to MD Trani, 56% of HCWs in Italy showed EE (Di Trani et al., 2021). In Portugal, Duarte I et al. concluded that more than half of HCWs had symptoms of personal burnout (Duarte et al., 2020). In Africa, owing to both the COVID-19 pandemic and civil war, 67.1% of Libyan HCWs reported having EE (Elhadi et al., 2020). Although the relationship between burnout symptoms and the COVID-19 pandemic has been proved, further research about the risk factors and how to alleviate burnout symptoms among frontline HCWs fighting against COVID-19 is still needed.

HCWs often work under great pressure and experience negative emotions owing to the nature of their work (Huo et al., 2021). Against the background of the COVID-19 outbreak, HCWs have faced more pressure than usual, every frontline HCW has faced enormous pressure. A recent study has reconfirmed the negative impact of the long-lasting pandemic in Saudi Arabia among healthcare students (Shaikh et al., 2021). HCWs were exposed to increased pressure, excessive workloads, extended work hours (Huo et al., 2021), and sleep deprivation—all of which are well-documented factors leading to burnout (Wang et al., 2005; Németh, 2016). Under the extraordinary conditions of the pandemic, HCWs have had insufficient protection against the disease and an increased risk of nosocomial infection (Centers for Disease Control and Prevention, 2008). Scholars have demonstrated the relationship between insufficient protection and anxiety and depression among HCWs during the COVID-19 pandemic (Pouralizadeh et al., 2020; Cag et al., 2021). The heavy workload, disruption of quotidian routines, and risk of COVID-19 infection experienced by HCWs owing to the pandemic all negatively affect the health of frontline HCWs. Chinese HCWs rose to the challenge, they grappled with the epidemic at the front line across the country, during the pandemic. However, to the best of our knowledge, scant research has specifically focused on both the known factors of burnout, such as excessive workload, and the factors directly associated with COVID-19 among Chinese HCWs. Therefore, the relationship between factors related to COVID-19 and burnout still needs to be explored in China.

Burnout is related to a wide range of harmful health outcomes, such as insomnia (Salvagioni et al., 2017), depression (Lu et al., 2020), loss of enthusiasm for work (Christian, 2015), decreased job satisfaction (Khamisa et al., 2015), resignation and early retirement (Khan et al., 2018), an increased risk of suicide (Johnson et al., 2018), and death by overwork (Yang et al., 2019). Most countries are currently under the double pressure of economic recovery and COVID-19 control, and thus must confront numerous challenges. For example, many governments have been reimposing and subsequently lifting restrictions according to new developments of the coronavirus disease. At the time of writing, new variants of COVID-19 have caused surges of the disease in the United Kingdom (Kirby, 2021). The Chinese government has implemented strategies for the ongoing prevention and control of COVID-19, as sporadic cases of the disease on the mainland have continued to be reported (The State Council Information Office of the People’s Republic of China, 2020). As HCWs worldwide need to not only perform their routine tasks, but also be alert to the danger of COVID-19, they are continually encountering the risk factors for burnout, and thus it is critical to alleviate HCWs’ burnout symptoms to maintain their mental and physical health, which Ali, S. et al. posited is a prerequisite for conquering the pandemic (Ali et al., 2020a). HCWs are on the frontline of this battle and are the last line of defence against COVID-19.

In this study, we aim to contribute to the knowledge of health systems worldwide by determining the prevalence and risk factors for burnout syndrome among frontline HCWs battling COVID-19 in China. Our study was conducted during an extraordinary period of the pandemic, in the time periods categorized by the Chinese government as Stage II (Initial Progress in Containing the Virus) and Stage III (Newly Confirmed Domestic Cases on the Chinese Mainland Drop to Single Digits) (The State Council Information Office of the People’s Republic of China, 2020). We propose that effective methods for alleviating burnout symptoms could be obtained by exploring the risk factors associated with burnout among frontline HWCs. Our research findings provide healthcare professionals and policy makers implications for the future surges of COVID-19 in a country or region. This study is valuable as we examine not only the known factors of burnout, such as workload, excessive working hours, diet, and sleep deprivation, but also the specific factors associated with COVID-19, such as HCWs’ infection protection and infection status.

## Materials and Methods

A cross-sectional survey was conducted among COVID-19 frontline HCWs in China from 1 February 8 to March 4, 2020, as China was going through Stage II and Stage III of the pandemic (The State Council Information Office of the People’s Republic of China, 2020). Our study’s participants were selected through cluster sampling. We identified three cities—Wuhan, Harbin, and Shenzhen—representing areas which were severely impacted by COVID-19. We used the formula:N=4⁢μα2⁢S2/δ2. The necessary sample size was 246 for each city, and after considering issues related to questionnaire recovery and efficiency, we added 10% to this total sample size. Ultimately, the minimum necessary sample size was 273 for each city. Our survey targeted three hospitals in each city, and we communicated with the hospital deans directly *via* personal contacts or email. We clarified significance and importance of our study to deans, if they indicated the willingness to join our study, deans of hospitals clarified significance and importance of our study to HCWs, frontline HCWs who were working in the departments related to COVID-19 were invited to participate in the survey. Finally, we recruited participants through eight hospitals—three in Wuhan, three in Harbin, and two in Shenzhen.

**FIGURE 1:**
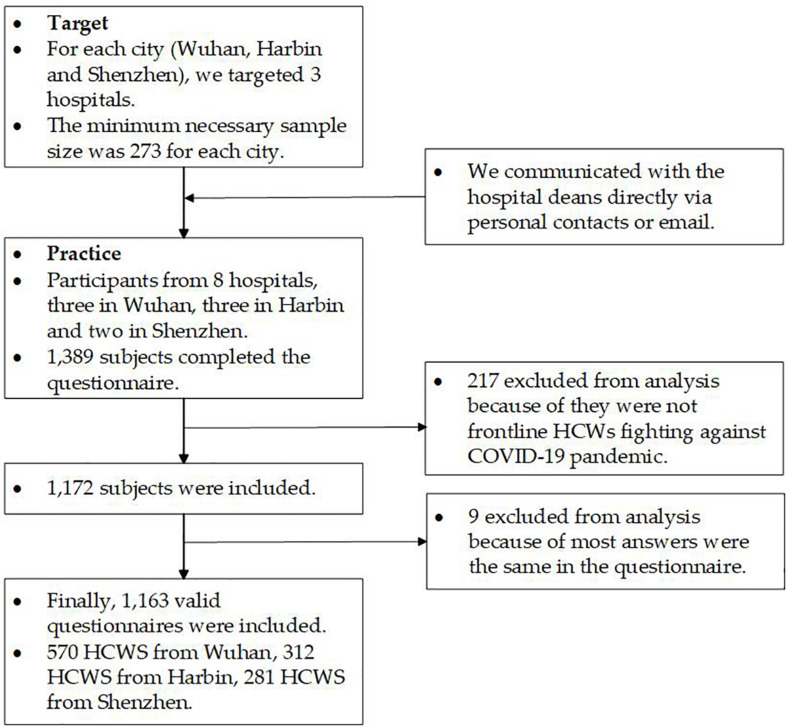
Study flow diagram.

HCWs answered the questionnaires during their breaks from work. Only completed questionnaires can be submitted. A total of 1,389 subjects completed the questionnaire. As the inclusion criterion was HCWs on the COVID-19 frontlines, the respondents were asked to answer the question, “Are you a frontline healthcare worker who is working for patients with COVID-19, such as diagnosing, treating, or nursing patients with COVID-19?”; 217 respondents were excluded from the survey. The research team reviewed the questionnaires, and excluded questionnaires in which most answers were the same; nine questionnaire respondents were excluded. Finally, 1,163 valid questionnaires were included (effective rate = 83.73%), among them 570 HCWs from Wuhan, 312 HCWs from Harbin, and 281 HCWs from Shenzhen. The participants were all recruited from capital cities and worked in tertiary public hospitals (Figure 1).

We received approval from the ethics committee of Harbin Medical University to conduct this study (Harbin, Heilongjiang, China), ID: HMUIRB20200003.

Our instrument comprised items on participants’ demographic information, the Maslach Burnout Inventory (MBI) scale, work situation, diet and sleep patterns, status of infection protection, status of COVID-19 infection of themselves and their colleagues, and status of psychological crisis intervention.

The basic socio-demographic information included age, gender, and job category. The job category was grouped into four types: nurses, doctors, technicians, and administrators.

Burnout among participants was assessed using the MBI scale. The MBI scale contains 22 items (Maslach et al., 1996). In this study, we used the Chinese version revised by Li and Liu (2000). The subscales included emotional exhaustion (EE, 9 items), depersonalisation (DP, 5 items), and personal accomplishment (PA, 8 items). All items were scored on a seven-point Likert scale, ranging from 0 (*never*) to 6 (*every day*). As in previous studies and according to convention, burnout was defined as high EE (scores of 27 or greater) and/or high DP (scores of 10 or greater) as opposed to a total score (Maslach and Jackson, 1981; Rotenstein et al., 2018; West et al., 2018). Higher scores on the EE and DP subscales indicate a higher burnout symptom, while PA was inversely associated with burnout (Gan et al., 2019; Brady et al., 2020). The low PA defined as scores of 33 or lower (Bourne et al., 2019). HCWs were categorised as having a high level of burnout if they scored high on EE and DP and low on PA (Gan et al., 2019).

In the reliability and validity analysis of MBI scale, the first point to consider about variables is internal consistency reliability. Cronbach’s α for the whole scale was 0.930; 0.936 for the EE subscale; 0.859 for the DP subscale; and 0.877 for the PA subscale; all of which demonstrated a high level of reliability. The second point to consider about variables is validity. The validity check is done in two stages as convergent validity and discriminant validity. The composite reliability (CR) and the explained average variance extracted (AVE) values were considered for convergent validity. The discriminant validity of all constructs met the Fornell and Larcker criteria (Fornell and Larcker, 1981). Table 1 presents a summary of the factor loadings, CR, AVE, and convergence validity. The standardized factor loadings of items are between 0.552 and 0.863, with good item reliability. The CR values of the 3 constructs range from 0.855 to 0.924, exceeding 0.7 (Hair et al., 2011). The AVE value of EE and DP constructs were higher than the threshold of 0.5, and the AVE value of PA constructs was closed to 0.5, which confirms the constructs’ convergent validity (Ye et al., 2019). Also, X^2^/df = 7.673, the comparative fit index = 0.927, the Tucker-Lewis fit index = 0.910, and the root mean square error of approximation = 0.076. The MBI scale showed acceptable reliability and validity (Table 1).

**TABLE 1:** Convergent validity and discriminant validity.

	**STD**	**CR**	**AVE**	**EE**	**DP**	**PA**
EE	0.624–0.844	0.924	0.576	0.753		
DP	0.591–0.814	0.855	0.545	0.717	0.738	
PA	0.552–0.863	0.866	0.452	0.102	-0.010	0.672

*STD, standardised factor loadings; CR, composite reliability; AVE, average variance extracted.*

Participants’ work situations were assessed with one open-ended question: “How many hours do you work every day?”.

Participants’ diet and sleep patterns were assessed with one item: “Are you able to eat three regular daily meals?” to which they answered yes or no, and two open-ended questions: “How many millilitres (ml) of water do you drink every day?” and “How many hours did you sleep per day in the past week?”

Participants’ status of infection protection were assessed with two items: “Could you adhere to infection control procedures?” and “Are you satisfied with the hospitals’ infection control measures?” to which they answered yes or no.

The status of COVID-19 infection of participants and their colleagues were assessed with two items: “Have you ever been infected with COVID-19?” and “Have your colleagues ever been infected with COVID-19?” to which they answered yes or no.

The status of psychological crisis intervention were assessed with one item: “Could you receive sufficient psychological crisis intervention?” to which they answered yes or no.

### Statistical Analyses

IBM^®^ SPSS^®^ Statistics 25.0, Mplus Version 7.0 and STATA 16.0 were used for the data analysis in this study. Heterogeneity analysis were computed using STATA 16.0. Heterogeneity were assessed using the I^2^ statistic. We explored the heterogeneity of age, gender, EE, DP, and PA among Wuhan, Harbin and Shenzhen, the results showed that there was no statistical significance between age (*I*^2^ = 0.0%, *P* = 0.965), gender (*I*^2^ = 0.0%, *P* = 0.990), EE(*I*^2^ = 0.0%, *P* = 0.983), DP(*I*^2^ = 0.0%, *P* = 0.982), and PA (*I*^2^ = 0.0%, *P* = 0.827) and location. Validity of MBI scale was tested using MplusVersion 7.0 (Muthén and Muthén, 1998–2012). The *t*-test, ANOVA test, and multiple linear regression were tested using IBM SPSS Statistics 25.0. The *t*-test and ANOVA test were performed to assess whether the independent variables were statistically significant. Stepwise multiple linear regression analysis was used to estimate the predictors of HCWs’ burnout and its three subscales. The significance level was set at 0.05.

## Results

### Burnout Classifications for Our Sample

The analysis revealed that burnout is widespread. High EE was found in 434 participants (37.3%), 466 participants (40.1%) showed high DP, and 750 participants (64.5%) showed low PA. Of the participants, 565 (48.6%) exhibited burnout. Of these 565 participants, 254 (21.8% of all participants) showed a high degree of burnout.

### Participant Characteristics and MBI Score

#### Participant Demographic Characteristics and MBI Score

The number of missing responses and items that were answered with “not applicable” are shown in Table 1 and ranged from 0.8 to 2.1%. Characteristics of subjects and distributions of each dimension of burnout in categorical items are also shown in Table 1. Among the participants, 72.3% were female and 27.7% were male, and 45.7% were older than or aged 35. Mean PA differed between age groups (*t* = −2.030, *P* = 0.043). Of these, 55.2% were nurses, 27.0% were doctors, 10.7% were technicians, and 7.1% were administrators. Mean EE (*F* = 6.741, *P* = 0.000), mean DP (*F* = 7.103, *P* = 0.000), and mean PA (*F* = 4.178, *P* = 0.006) differed between job category groups. Doctors had higher EE scores and higher DP scores than administrators (Table 2).

**TABLE 2:** Descriptive statistics and univariate analysis results.

		**EE**	**DP**	**PA**
	**N(%)**	**Mean (SD)**	**Mean (SD)**	**Mean (SD)**

**Demographic characteristics and MBI score**				
**Gender**				
female	841 (72.3)	21.3 (13.1)	7.9 (6.5)	29.7(9.9)
male	322 (27.7)	21.0 (13.3)	8.4 (6.9)	29.4 (10.5)
F/t		0.367	1.200	0.470
**Age (yr)**				
≤34	632 (54.3)	21.3 (13.0)	8.3 (6.7)	29.1 (10.0)
≥35	531 (45.7)	21.1 (13.2)	7.8 (6.6)	30.3 (10.2)
F/t		0.304	1.088	–2.030*
**Job category**				
Nurse	642 (55.2)	20.4 (12.6)	7.8 (6.4)	30.3 (9.8)
Doctor	314 (27.0)	23.9 (13.4)	9.4 (6.6)	29.6 (9.3)
Technician	125 (10.7)	20.3 (14.0)	6.7 (6.9)	28.5 (11.9)
Administrator	82 (7.1)	18.5 (13.3)	7.0 (7.3)	26.4 (11.2)
F/t		6.741***	7.103***	4.178**

**Work characteristics and MBI score**				
**Work more than 8 consecutive hours a day**				
No	840 (72.2)	19.8 (12.8)	7.7 (6.5)	29.7 (10.3)
Yes	299 (25.7)	25.0 (13.1)	9.2 (6.9)	29.5 (9.4)
Missing	24 (2.1)			
F/t		5.852***	–3.396***	0.312
**Working in Wuhan when they answered the questionnaire**				
No	593 (51.0)	22.0 (13.8)	8.6 (6.9)	28.7 (10.6)
Yes	570 (49.1)	20.4 (12.4)	7.5 (6.3)	30.6 (9.5)
F/t		1.962*	3.055**	–3.244***

**Diet, sleep pattern, and MBI score**				
**Three regular meals a day**				
Be able to eat	849 (73.0)	20.4 (12.7)	7.8 (6.4)	29.8 (10.0)
Unable to eat	314 (27.0)	23.3 (14.1)	8.8 (7.1)	29.2 (10.2)
F/t		–3.150***	–2.262*	0.828
**Daily water intake**				
≤800ml	586 (50.4)	23.2 (13.4)	8.6 (6.8)	29.0 (10.0)
>800ml	552 (47.5)	19.0 (12.4)	7.4 (6.3)	30.5 (10.1)
Missing	25 (2.1)			
F/t		–5.563***	–3.235**	2.519*
**Daily sleep hours**				
≤6 h	657 (56.5)	22.6 (13.3)	8.2 (6.8)	30.3 (10.0)
>6 h	497 (42.7)	19.4 (12.7)	7.8 (6.4)	28.9 (10.2)
Missing	9 (0.8)			
F/t		–4.106***	1.054	–2.375*

**Infection protection and MBI score**				
**Adhering to infection control procedures**				
No	89 (7.7)	30.1 (12.9)	11.4 (6.7)	26.0 (7.5)
Yes	1074 (92.3)	20.5 (12.9)	7.8 (6.6)	29.9 (10.2)
F/t		6.811***	4.953***	–4.634***
**Hospital’s infection control measures**				
Dissatisfied	195 (16.8)	26.8 (14.7)	9.9 (7.0)	29.2 (10.2)
Satisfied	968 (83.2)	20.1 (12.5)	7.7 (6.5)	29.7 (10.1)
F/t		5.962***	4.232***	–0.583

**Infection status and MBI score**				
**Who were infected or had colleagues who were infected with COVID-19**				
No	946 (81.3)	20.2 (13.0)	7.7 (6.5)	29.6 (10.3)
Yes	217 (18.7)	25.5 (12.7)	9.7 (7.0)	29.7 (9.2)
F/t		–5.356***	–4.054***	–0.159

**Psychological crisis intervention and MBI score**				
**Receive sufficient psychological crisis intervention**				
No	632 (54.3)	22.9 (13.3)	8.6 (6.6)	28.6 (10.0)
Yes	531 (45.7)	19.2 (12.6)	7.4 (6.6)	30.8 (10.1)
F/t		4.938***	3.265***	–3.639***

*p ≤ 0.05; **p ≤ 0.01; ***p ≤ 0.001.

#### Work Situations and MBI Score

Of the participants, 25.7% worked more than 8 consecutive hours a day and showed higher EE scores (*t* = −5.852, *P* = 0.000) and higher DP scores (*t* = −3.396, *P* = 0.001). Nearly half of the participants (49.1%) were working in Wuhan when they answered the questionnaire and showed lower EE scores (*t* = 1.962, *P* = 0.050), lower DP scores (*t* = 3.055, *P* = 0.002), and higher PA scores (*t* = −3.244, *P* = 0.001) (Table 2).

#### Diet, Sleep Patterns, and MBI Score

A total of 27.0% of the participants expressed that they were unable to eat three regular daily meals and showed higher EE scores (*t* = −3.150, *P* = 0.001) and higher DP scores (*t* = −2.262, *P* = 0.018). A total of 50.4% of the participants drank no more than 800 ml of water every day and showed higher EE scores (*t* = −5.563, *P* = 0.000), higher DP scores (*t* = −3.235, *P* = 0.010), and lower PA scores (*t* = 2.519, *P* = 0.012). The average amount of sleep obtained by participants was 6–1/2 h per day in the past week while 56.5% slept for no more than 6 h per day and showed higher EE scores (*t* = −4.106, *P* = 0.000) and higher PA scores (*t* = −2.375, *P* = 0.018) (Table 2).

#### Infection Protection and MBI Score

A total of 7.7% of the participants reported that they could not adhere to infection control procedures and showed higher EE scores (*t* = 6.811, *P* = 0.000), higher DP scores (*t* = 4.953, *P* = 0.000), and lower PA scores (*t* = −4.634, *P* = 0.000) than those who could adhere to infection control procedures. Among the participants, 16.8% were “dissatisfied” and 83.2% were “satisfied” with their hospital’s infection control measures. Participants who reported “dissatisfied” showed higher EE scores (*t* = 5.962, *P* = 0.000) and higher DP scores (*t* = 4.232, *P* = 0.000).

#### Status of COVID-19 Infection and MBI Score

A total of 946 participants (81.3%) reported that neither themselves nor their colleagues were infected while 217 participants (18.7%) reported that they or their colleagues were infected. Among the 217 participants, 65 (5.6%) reported that they were infected, 213 (18.3%) reported that their colleagues were infected, and 61 (5.2%) reported that both themselves and their colleagues were infected. Participants who reported that they or their colleagues were infected showed higher EE scores (*t* = −5.356, *P* = 0.000) and higher DP scores (*t* = −4.054, *P* = 0.000) (Table 2).

#### Psychological Crisis Intervention and MBI Score

Of the participants, 54.3% reported that they did not receive sufficient psychological crisis intervention and showed higher EE scores (*t* = 4.938, *P* = 0.000), higher DP scores (3.265, *P* = 0.001), and lower PA scores (*t* = −3.639, *P* = 0.000) than those who expressed that they did receive sufficient psychological crisis intervention (Table 2).

### Workload of Participants

Participants worked an average of 8.1 ± 4.2 h per day. The average working hours for nurses, doctors, technicians, and administrators were 6.7, 9.9, 9.6, and 9.6 h per day, respectively (*F* = 57.249, *P* = 0.000) (Table 3).

**TABLE 3:** Average working hours for participants with different characteristics.

	**N (%)**	**Work hours (SD)**
Job category		*F* = 57.249, *P* = 0.000
Nurse	627 (55.0)	6.7 (3.5)
Doctor	308 (27.0)	9.9 (4.5)
Technician	122 (10.7)	9.6 (4.5)
Administrator	82 (2.1)	9.6 (3.5)
Missing (daily work time)	24 (2.1)	

### Factors Associated With Burnout in the Multiple Linear Regression Model

Participants who were nurses (*b* = 3.067, *P* = 0.042), who were doctors (*b* = 3.954, *P* = 0.011), who were working continuously for more than 8 h a day (*b* = 3.392, *P* = 0.000), who were unable to eat three regular daily meals (*b* = 2.225, *P* = 0.008), whose daily water intake was no more than 800 ml (*b* = 3.007, *P* = 0.000), who obtained no more than 6 h of sleep a day (*b* = 1.609, *P* = 0.036), and who were infected or had colleagues who were infected with COVID-19 (*b* = 4.182, *P* = 0.000) were more likely to experience high EE. Participants who could adhere to infection control procedures (*b* = −5.992, *P* = 0.000), who were satisfied with their hospital’s infection control measures (*b* = −3.709, *P* = 0.001), and who could receive sufficient psychological crisis intervention (*b* = −1.588, *P* = 0.039) were more likely to experience lower EE (Table 4).

**TABLE 4:** Multiple linear regression analysis results for EE, DP, and PA.

	**EE**				**DP**				**PA**			
**Variable**	**B**	**SE**	**Beta**	**p**	**B**	**SE**	**Beta**	**p**	**B**	**SE**	**Beta**	**p**
			**standardized**				**standardized**				**standardized**	
Age(<34-ref)									1.859	0.655	0.092	0.005**
Job category (Administrator-ref)												
Nurse	3.067	1.508	0.117	0.042*	1.390	0.786	0.105	0.077	3.529	1.249	0.155	0.005**
Doctor	3.954	1.547	0.134	0.011*	1.990	0.807	0.134	0.014*	4.147	1.245	0.204	0.001***
Technician	0.398	1.784	0.009	0.823	–1.228	0.930	–0.057	0.187	2.652	1.446	0.080	0.067
Working in Wuhan (No-ref)	–0.198	0.846	–0.008	0.815	–1.247	0.440	–0.094	0.005**	1.377	0.643	0.068	0.033*
Work continuously >8 h a day (No-ref)	3.392	0.928	0.114	0.000***	0.775	0.475	0.051	0.104	0.151	0.747	0.007	0.840
Unable to eat three regular daily meals (Able to eat three regular daily meals-ref)	2.225	0.841	0.075	0.008**	0.987	0.437	0.066	0.024*				
Daily water intake ≤ 800ml (> 800ml-ref)	3.007	0.754	0.115	0.000***	0.713	0.392	0.054	0.069	–1.145	0.606	-0.057	0.059
Daily sleep hours ≤6 h (> 6h-ref)	1.609	0.767	0.061	0.036*					1.848	0.621	0.091	0.003**
Adhering to infection control procedures (No-ref)	–5.992	1.461	–0.123	0.000***	–2.288	0.762	–0.093	0.003**	3.210	1.140	0.085	0.005***
Satisfied with hospitals’ infectious control measures (Dissatisfied-ref)	–3.709	1.062	–0.105	0.001***	–0.908	0.554	–0.051	0.101				
Who were infected or had colleagues who were infected with COVID-19 (No-ref)	4.182	1.000	0.125	0.000***	1.974	0.520	0.117	0.000***				
Receive sufficient psychological crisis intervention (No-ref)	–1.588	0.767	–0.061	0.039*	–0.572	0.399	–0.043	0.152	1.569	0.616	0.077	0.011*
Constant coefficient	23.016	2.123		0.000	9.373	1.081		0.000	20.435	1.687		0.000
	*R*^2^ = 0.137, Adjust *R*^2^ = 0.128				*R*^2^ = 0.080, Adjust *R*^2^ = 0.070				*R*^2^ = 0.051, Adjust *R*^2^ = 0.043			

B = unstandardised regression coefficient; SE = standard error. *p ≤ 0.05; **p ≤ 0.01; ***p ≤ 0.001.

Participants who were doctors (*b* = 1.990, *P* = 0.014), who were unable to eat three regular daily meals (*b* = 0.987, *P* = 0.024), and who were infected or had colleagues who were infected with COVID-19 (*b* = 1.974, *P* = 0.000) were more likely to experience higher DP. Participants who were working in Wuhan (*b* = −1.247, *P* = 0.005) and who could adhere to infection control procedures (*b* = −2.288, *P* = 0.003) were more likely to experience lower DP (Table 4).

Participants who were aged ≥35 (*b* = 1.859, *P* = 0.005), who were nurses (*b* = 3.529, *P* = 0.005), who were doctors (*b* = 4.147, *P* = 0.001), who were working in Wuhan (*b* = 1.377, *P* = 0.033), who got no more than 6 h of sleep a day (*b* = 1.848, *P* = 0.003), who could adhere to infection control procedures (*b* = 3.210, *P* = 0.005), and who could receive sufficient psychological crisis intervention (*b* = 1.569, *P* = 0.011) were more likely to experience higher PA (Table 4).

## Discussion

Frontline HCWs battling COVID-19 exhibited a high level of burnout in China. According various studies, the prevalence of burnout in HCWs in Asia during the COVID-19 pandemic varies from 31.4 to 75% (Khasne et al., 2020; Matsuo et al., 2020; Alsulimani et al., 2021; Huo et al., 2021). The large differences across these studies may result from regional disparities and variations in burnout definitions and assessment methods (Rotenstein et al., 2018). In our study, 48.6% of frontline HCWs were suffering from burnout, higher than the figure (36.5%) reported in a previous study conducted among Chinese HCWs during the pandemic (Huo et al., 2021). The prevalence of EE symptom (37.3%) is higher than that (34.2%) reported in another study conducted in Wuhan Jinyintan Hospital (Li et al., 2021).

The most important findings of the present study are the following: infection of HCWs, long continuous working hours, inability to eat three regular daily meals, insufficient water intake, and insufficient sleep increase burnout. Conversely, adherence to infection control procedures, satisfaction with their hospital’s infection control measures, and sufficient psychological crisis intervention could decrease burnout.

In this study, nurses and doctors who were working in Wuhan when they answered the questionnaire showed a lower level of DP and a higher level of PA in the multiple linear regression analysis. In other words, they showed less negative attitudes toward their job and workplace and higher personal accomplishment (Mealer et al., 2016; Bridgeman et al., 2018). During the Stage I (Swift Response to the Public Health Emergency), HCWs in Wuhan worked under great pressure, which attracted much attention of Chinese government and members of society. Later, from 23 January to 08 March 2020, more than 42,600 HCWs across China were dispatched to Hubei Province, especially Wuhan. The workload of HCWs in Hubei Province was alleviated by 346 medical teams from around the country (The State Council Information Office of the People’s Republic of China, 2020), as these additional human resources facilitated better work schedules and shorter work shifts. Local HCWs of Wuhan formed support groups with other HCWs from around the country, which provided HCWs further relief in terms of moral support. In addition to this psychological support, hospitals in Wuhan received material support from across China, such as rice, vegetables, meals, and personal protective equipment (PPE). Recent studies revealed that having resources such as social support, sufficient material, and adequate human resources (staff) correlate negatively with burnout (Algunmeeyn et al., 2020; Manzano García and Ayala Calvo, 2021). These measures could explain why HCWs in Wuhan had lower levels of DP and higher levels of PA.

People most at risk of infection are those who are in close contact with a COVID-19 patient or who care for COVID-19 patients (World Health Organization, 2020). Participants who indicated that they could adhere to infection control procedures showed lower levels of EE, lower levels of DP and higher levels of PA. Participants who indicated that they were satisfied with their hospital’s infection control measures showed lower levels of EE. Participants who reported that they or their colleagues were infected showed higher levels of EE and DP. Due to the suddenness of the outbreak, many HCWs did not have what they need to treat patients and they could not adhere to infection control procedures, which would lead to a risk of infection for HCWs (Ali et al., 2020b; Wang et al., 2020). Together with the fear of passing the virus on to their families and friends (Adams and Walls, 2020; Chen et al., 2020; Dong et al., 2020), HCWs’ concerns about having insufficient protection against COVID-19 causes negative emotional reactions. Recent studies found that having insufficient protection inflicts considerable mental damage on HCWs, such as anxiety and depression (Pouralizadeh et al., 2020; Cag et al., 2021). The results of our study showed that insufficient protection is one of the predictor variables of burnout symptoms among HCWs.

To protect HCWs, Chinese healthcare professionals and policymakers took various measures. First, the Chinese government stipulated comprehensive incentive plans to encourage production enterprises to accelerate production. Second, more stringent and comprehensive hospital infection control measures were implemented. On February 19, 2020, the National Health Commission issued a directive on strengthening the protection of HCWs (National Health Commission of the People’s Republic of China, 2020), and national, provincial, municipal, and local levels of expert committees on hospital infection control and treatment were established, resulting in the formulation and implementation of various protocols and procedures.

Due to the COVID-19 outbreak, the normal routine of HCWs was severely disrupted. First, they had to face increased workloads and insufficient rest. During the early stages of the outbreak, there were Chinese news reported that HCWs worked continuously for long time, sometimes exceeding 14 h (SINA Corporation, 2020). In our study, 25.7% of the participants worked for more than 8 consecutive hours a day. The average working hours for doctors were 9.9 h a day and for technicians, such as medical imaging doctors, were 9.6 h a day. Nurses had the shortest daily working hours (6.7 h) which was due to the fact that they provided direct care for COVID-19 patients and their occupational protective clothing could only maintain effective protection for 4–6 h. HCWs experienced higher levels of EE when they worked continuously for more than 8 h a day, which is consistent with previous research that found that HCWs’ long working hours were associated with higher levels of anxiety, despair, and burnout (Elmore et al., 2016; Çelmeçe and Menekay, 2020). Previous studies have reported that as workload increases, HCWs have less time to recover from stressful situations, which will lead to increased rates of EE (Bridgeman et al., 2018). Our study found that during the COVID-19 outbreak, frontline HCWs’ heavy workload and lack of rest breaks may lead to burnout symptoms (Bridgeman et al., 2018). To reduce the continuous work periods of frontline HCWs, Chinese hospital administrators attempted to establish a more rational work shift system that would allow frontline HCWs to have rest breaks and to regularly alternate high-pressure roles with other HCWs, and strictly implemented the new work shift system to reduce job intensity and workload (Kang et al., 2020).

Second, irregular meals are a risk factor for burnout symptoms. Of the participants, 27.0% indicated that they were unable to eat three regular daily meals and they showed higher EE and DP, which was consistent with a previous study in which nurses believed they experienced burnout because of skipped or shortened lunches (Russell, 2016). Many Chinese HCWs were unable to eat three regular daily meals, owing to excess work, limited access to food, and other reasons during the pandemic. In the earlier stages of the pandemic in Wuhan, food resources were limited, and three regular daily meals could not be guaranteed to HCWs. In Wuhan, to meet the dietary needs of HCWs and patients, governments worked together with enterprises, social groups, and volunteers. They purchased ingredients, recruited transport vehicles, and transported free meals and edible to hospital canteens. Providing nutritious and regular meals to HCWs to ensure that they have sufficient energy to prevent burnout is an important intervention to alleviate burnout—and should be considered the responsibility of governments, hospitals, and society.

Third, many HCWs’ daily water intake was insufficient. Of the participants, 50.4% drank no more than 800 ml a day and showed higher EE. According to Atay S et al., in Turkey, nurses cared for patients with COVID-19 reported the most severe problem was perspiration when wearing overalls/gowns (84.1%) (Atay and Cura, 2020). Another study developed North India showed that extreme sweating (59.6%) was a serious problem among frontline nurses (Jose et al., 2021). Heavy work with insufficient water intake, water loss, and mild levels of dehydration can produce disruptions in mood and cognitive functioning, which can easily cause physical fatigue (Popkin et al., 2010). Hospital administrators should install more water dispensers in the right places, keep water safe, and encourage HCWs to drink more water during their breaks.

Forth, sleep deprivation and circadian disorders are inherent occupational risks for burnout of HCWs (Stewart and Arora, 2019). The results of our study showed that the risks of sleep deprivation were noticeable among Chinese HCWs during the pandemic. Of the participants, 56.5% slept for no more than 6 h a day and experienced higher levels of EE. In Wuhan, to ensure that HCWs had more time to rest, hospitals arranged for HCWs to stay in hotels or hospital dormitories as close to their workplaces as possible. This practice gave HCWs some time to have rest and reduced HCWs’ concerns that they would pass the virus to their families and friends.

Psychological status plays a major role in physicians’ mental well-being (Elhadi et al., 2021). Given the sweeping mental health impact of COVID-19, protecting HCWs from the adverse psychological effects of the pandemic is critical (Albott et al., 2020). In our study, 45.7% of the participants reported that they received sufficient psychological crisis intervention and they showed lower EE and higher PA. Previous study has warned that psychological interventions targeting HCWs are urgently needed (Luo et al., 2020). Comprehensive psychological intervention should be carried out in the pandemic. First, at the national level, psychological crisis intervention should occupy a pivotal place in the overall deployment of COVID-19 controls (Li et al., 2020). For example, following The National Health Commission of China call for psychological crisis intervention programs, expert teams were established to compile guidelines, and mental health professionals were stationed in designated isolation hospitals to provide on-site services. Second, at the organizational level, effective psychological crisis interventions that could contribute to the reduction of burnout, such as short-term counselling (Rø et al., 2008), Balint groups which is a group training method (Huang et al., 2020), and psychological screening should be employed during the workday in the disease outbreak period (Liu Z. et al., 2020). Third, at the personal level, HCWs should attend to their physiological health and practice self-care for nutrition, rest, and sleep.

The relationship between gender and burnout is also somewhat controversial (Sanfilippo et al., 2017; Low et al., 2019), in our study, the gender variable was not an influencing factor of burnout in multiple linear regression analysis, which is similar to the results of studies developed in China during the COVID-19 outbreak (Liu X. et al., 2020; Li et al., 2021). In our study, HCWs who were older showed higher PA, which was similarity to a study conducted among HCWs in China (Huo et al., 2021). That may be because during the pandemic, compared to power factors such as infection control, the influence of the gender variable and age variable was masked.

Our research showed that the overwhelming demand for the care of COVID-19 patients placed unprecedented burdens on HCWs, resulting in burnout. Our study identified the following risks for burnout. First, a heavy workload will increase the prevalence of burnout among HCWs. Second, insufficient protection against COVID-19 and the infection status of HCWs are risk factors for burnout. Third, the disruption of normal daily routines is harmful to HCWs, including working for more than 8 continuous hours; obtaining no more than 6 h of sleep; irregular meals; and limited water intake. Fourth, the lack of timely psychological crisis intervention is a risk factor for burnout. Therefore, a synergized and comprehensive interventions should be developed by the government and hospital administrators to address the burnout of frontline HCWs and to fight the COVID-19 pandemic. First, government should re-route critical resources such as workforces and PPE to hospitals that were hit the hardest, as the Chinese government did when it sent additional medical teams to Wuhan. Second, forceful interventions should be developed by government and hospital administrators to protect HCWs from infection. Third, structural or organizational interventions, such as workload or work shift rotation should be performed to allow frontline HCWs to alternate high-pressure roles with other HCWs, and also guarantee them to receive three regular daily meals, sufficient water, and sufficient sleep. Last, psychological interventions targeting HCWs are urgently needed.

This study has several limitations. First, the data are cross-sectional, a causal relationship could not be confirmed. Second, we were limited to an online anonymous questionnaire, which may provide an over- or under-estimation of the responses. However, the online questionnaire was the safest method of data collection as face-to-face communication was risky during this period. Third, the study covered the period, February 18 to March 04, 2020, which was over a year ago. However, since the study was conducted during a pivotal period of the fight against COVID-19 in China and the period is unrepeatable, our research results are valuable to understanding the alleviation of burnout symptoms of HCWs against the background of the surge of unknown infectious diseases. Forth, data were collected from participants’ self-reports; thus, inherent bias was unavoidable. Fifth, in order to not consume too much of HCWs’ time, our study did not involve other psychological evaluations. Sixth, the survey did not cover all the related factors for burnout in health professionals. We designed the questionnaire according to practical situation of China, and due to the period we designed the questionnaire, literatures related to burnout of HCWs among COVID-19 pandemic were lacking, maybe there were some factors that affect the results didn’t included in our study. Last, there were some innate recall bias in our study.

## Conclusion

In this study, we determined the prevalence of and risk factors for burnout syndrome among frontline HCWs fighting COVID-19. The results indicate that burnout—which is extremely harmful—is prevalent among frontline HCWs in the battle against COVID-19 in China. We found that infection of HCWs, long continuous working hours, inability to eat three regular daily meals, insufficient water intake, and insufficient sleep increase burnout. Conversely, adherence to infection control procedures, satisfaction with their hospital’s infection control measures, and sufficient psychological crisis intervention could decrease burnout. To alleviate the burnout symptoms of frontline HCWs, synergized and comprehensive interventions should be developed by governments and hospital administrators to address burnout. First, critical resource allocation should be prioritised in hospitals and workforces that are hit the hardest. Second, working in an unprotected environment, a lack of desperately needed PPE resources and infection status of HCWs may lead to burnout. Thus, governments and hospitals must protect HCWs from infection. Third, regarding hospital managers, a more rational workshift schedule should be strictly implemented to reduce the workload of those on the frontline. Fourth, hospital management measures, including the provision of nutritious and regular meals, sufficient drinking water, opportunity to sleep for more than 6 h, and more psychological interventions to counter burnout should be integrated into the coping strategy. In addition, psychological interventions targeting HCWs should be adopted at the national, organizational, and personal levels.

## Data Availability Statement

The raw data supporting the conclusions of this article will be made available by the authors, without undue reservation.

## Author Contributions

XZ, KW, and QW: conceptualization. XZ, MJ, NN, and ZK: formal analysis. KW, LS, WH, WY, and YW: investigation. XZ, LL, and LG: data curation. XZ, JW, and YH: writing—original draft preparation. WY and QW: writing—review and editing. All authors have read and agreed to the published version of the manuscript.

## Conflict of Interest

The authors declare that the research was conducted in the absence of any commercial or financial relationships that could be construed as a potential conflict of interest.

## Publisher’s Note

All claims expressed in this article are solely those of the authors and do not necessarily represent those of their affiliated organizations, or those of the publisher, the editors and the reviewers. Any product that may be evaluated in this article, or claim that may be made by its manufacturer, is not guaranteed or endorsed by the publisher.

## Abbreviations

COVID-19: Novel coronavirus disease
HCW: Healthcare worker
MBI scale: Maslach Burnout Inventory scale
EE: emotional exhaustion
DP: depersonalization
PA: personal accomplishment
PPE: personal protective equipment
CR: composite reliability
AVE: average variance extracted.
